# Investigating Resilience in Healthcare: Easier Said Than Done? A Response to the Recent Commentaries

**DOI:** 10.34172/ijhpm.2022.7682

**Published:** 2022-10-12

**Authors:** Andrew Smaggus, Janet C. Long, Louise A. Ellis, Robyn Clay-Williams, Jeffrey Braithwaite

**Affiliations:** ^1^Queen’s University, Kingston, ON, Canada.; ^2^Australian Institute of Health Innovation, Macquarie University, Sydney, NSW, Australia.

 It is a great pleasure to provide this response to the recent commentaries on our article “*Government Actions and Their Relation to Resilience in Healthcare During the COVID-19 Pandemic in New South Wales, Australia and Ontario, Canada*.^”1^ We wish to begin by expressing our appreciation to the authors for the time and effort they devoted to these commentaries. Each article adds to the scholarly conversation surrounding resilience in healthcare, and together they raise conceptual and methodological issues in need of attention. The quality of these commentaries will help the healthcare community construct more humane and resilient systems in the wake of the disruptions caused by the coronavirus disease 2019 (COVID-19) pandemic. In this correspondence, we will provide brief reviews of each commentary. Please note that the range and depth of analysis in each commentary precludes comprehensive summaries in this space. Instead, we will attempt to find common threads across the six articles. We will then conclude with an attempt to synthesize these threads and identify two overarching opportunities that researchers can address to advance the study of resilience in healthcare.

 To begin, Øyri and Wiig report on an investigation that looked for elements of resilience in the Norwegian government’s actions during the COVID-19 pandemic.^2^ The authors adopted a situated, structural, and systemic framework of resilience to identify the activities and adaptations made across system levels in Norway during the pandemic. Their analysis leads to conclusions that appear to align with our own: elements of resilience are identifiable throughout these actions, the capacity to learn is crucial to developing resilience, and there may be benefits to a broader appreciation of resilience and its tenets. They also make a significant advance on our work by recognizing the importance of innovation in fostering resilience (a point we will return to below). Øyri and Wiig conclude with the astute observation that capacities and circumstances vary at the micro, meso, and macro levels of a system. Therefore, understanding healthcare systems requires attention to these variations and how they affect events and perspectives within individual system levels.

 Anderson’s commentary addresses similar issues while providing a comprehensive overview of the many challenges facing researchers studying resilience in healthcare.^3^ Anderson acknowledges the challenges of finding methods conducive to analyzing the complex, non-linear relationships between elements and levels within healthcare systems. These methodological challenges are likely part of the reason that most healthcare research on resilience has focused on local, short-term adaptations. A fuller understanding of resilience requires expanding our methodological options to explore larger scales and longer periods of time (eg, how decisions made by current and previous governments establish the preconditions for existing capacities for resilient performance). Anderson emphasizes the importance of using multiple, complementary frameworks and methods (a shortcoming of our study that we acknowledge) to achieve these goals and fully appreciate the mechanisms that contribute to a system’s capacity for resilient performance.

 Aase adopts a more specific focus in her commentary by highlighting the difficulty of defining meaningful outcomes in resilience research.^4^ Again recognizing the complexity of healthcare systems, Aase calls attention to the goal conflicts that arise between different agents and groups occupying different perspectives within a system. What might appear to be a desirable outcome at one level of the system may compromise resilience at another level. The ambiguity of outcomes occurs not only across different levels but also across time. Today’s outcomes become the preconditions for tomorrow’s outcomes. Thus, what one considers an outcome is a matter of perspective, as are decisions regarding what constitutes a positive or negative outcome. While this diversity of perspectives is often a strength for the system, it can be challenging to handle for researchers. Therefore, it is not surprising that many studies of resilience in healthcare (including, unintentionally, our own) subtly evade the issue of outcomes. To address this issue, Aase agrees with Anderson in recommending that we embrace multiple frameworks and methods. This approach could allow researchers to capture as many different perspectives as possible in their efforts to construct meaningful outcomes while also recognizing that no single outcome can reflect the goals and values of all agents within a system.

 Angeler and colleagues use their commentary to present a strong case for a transdisciplinary approach to fostering and studying resilience.^5^ Within their argument, they note the dangers of “command and control” approaches that can restrict diversity of thought and contribute to an excessive focus on a single perspective or goal. These authors also provide stirring insights on the importance of “transformation” in creating resilience (echoing Øyri and Wiig’s comments on innovation). Transformation has received little attention in healthcare compared to that given to adaptive capacity at the frontlines. However, the individuals working in strained systems cannot absorb disturbances indefinitely (a point supported by the effects of pandemic-era stresses on healthcare workers’ mental health).^6^ At some point, systemic transformation is necessary. This issue, understood in socio-ecological approaches to resilience,^7,8^ reinforces the authors’ call for transdisciplinary collaboration and forms a component of the theoretical model they provide.

 Bozorgmehr and colleagues further the discussion of transformation as a component of resilience.^9^ To develop the transformative capacity of healthcare systems, the authors propose exploring how decision makers’ use of uncertainty (eg, whether governments frame the uncertainty of a pandemic with hope or with fear) shapes potential modes of transformation. Relationships and interconnections between elements and levels of a system also shape transformative capacity, which the authors believe reinforces the importance of understanding the nested nature of systems and the ways that different system levels interact with each other. The authors conclude with a call for clarity regarding the idea of resilience itself, noting that the concept is variably treated as an outcome, a mediator, or a determinant.

 Many of the issues discussed above come together in the commentary of Leistikow and Bal.^10^ These authors provide a measured (and much appreciated) critique of our original paper that serves as a platform for methodological guidance. This guidance reiterates the importance of accessing the multiple perspectives present within systems by utilizing multiple frameworks, methods, and data sources. The authors encourage a transdisciplinary approach by suggesting the benefits of insights from fields such as politics and public administration (eg, how the concept of path dependence could facilitate the analysis of temporal elements of resilience). With reference to our original study, they also provide a reminder of the need for direct observation of the micro and meso levels of healthcare to understand how macro-level actions impact other levels. In concert with the other commentaries, Leistikow and Bal begin to provide a picture of what ideal investigations of resilience in healthcare could look like going forward.

## Translating Guidance Into High-Quality Research

 These commentaries have consolidated a substantial amount of methodological guidance for studying resilience. In that respect, this collection of articles is a valuable resource for researchers of resilience in healthcare. But now comes the hard part. Researchers still face the challenge of applying this guidance to create concrete, high-quality studies. While this will be a difficult task, we believe these commentaries highlight two overarching opportunities that the resilience in healthcare community can address to enhance research in the field.

 The first opportunity involves developing greater conceptual clarity regarding resilience and its elements. Despite the recent progress in studying resilience in healthcare, the answers to several important conceptual questions remain ambiguous. Bozorgmehr and colleagues raise one such question: how should we classify resilience? As an outcome, a mediator, or a determinant? A more fundamental question to consider may be: what are we talking about when we talk about resilience? Are we talking about adaptive capacity at the frontlines (as often appears to be the case in resilience in healthcare studies)? About the capacity of systems to transform? About the ability to recover from events or about proactive risk management? Or are we talking about all of the above?

 These questions deserve further exploration and debate amongst resilience in healthcare scholars. Opinions are likely to differ (eg, some may feel that resilience does not fit exclusively in any one of the outcome, mediator, or determinant categories) and exploring those differences could help develop conceptual clarity. It may be the case that achieving a broad consensus on these questions is not possible (because, for example, one’s definition of resilience depends on one’s perspective). Even so, individual researchers may do well to consider these questions when designing their studies in order to achieve clarity on how they are using the concept of resilience.

 The second opportunity involves addressing the challenges associated with studying complex systems. Complex socio-technical systems have unique properties that make their behaviour difficult to comprehend.^11^ As mentioned by many commentators, the individuals and groups of a complex system inhabit different perspectives and possess different values, creating diversity throughout the system. Complex systems also evolve as individuals learn and change their behaviours based on what they learn.^12^ As a result, those studying these systems (as is the case for resilience in healthcare scholars) should seek to assess multiple perspectives over extended time frames. Achieving this goal with finite resources will require thoughtful methodological approaches that utilize multiple methods.

 Furthermore, studying complexity is an area where transdisciplinary approaches have the potential to enhance research on resilience in healthcare. While the study of complex systems remains relatively new within healthcare, other disciplines have more mature approaches to dealing with the challenges such systems present.^13-15^ Angeler and colleagues’ model provides ideas of fields and paradigms that could help to develop this aspect of resilience in healthcare.^5^ For example, ecological systems must be capable of adapting, evolving, and transforming to face emergent challenges. Ecologists have developed concepts (eg, panarchy^16,17^) that facilitate studying how these systems fulfill these requirements. These concepts have received little attention in healthcare and could represent an untapped resource to enhance our understanding of the systems in which we work.

## Conclusion

 This collection of commentaries and the guidance they contain will likely serve those researching resilience in healthcare well as the field moves into the future (in fact, we would have benefitted from access to these insights during our original study). However, we must also remember that these suggestions are likely easier said than done. The challenge of applying this guidance to the messiness of the real world remains, and advances may arrive slowly. Nevertheless, resilience researchers have made important contributions to conversations on how to enhance the provision of healthcare. If we are able to develop greater conceptual clarity (through scholarly debate) and attend to the challenges of studying complex systems (using thoughtful methodological approaches and transdisciplinary collaboration), these contributions are sure to grow and benefit healthcare systems around the world.

## Ethical issues

 Not applicable.

## Competing interests

 Authors declare that they have no competing interests.

## Authors’ contributions

 All authors contributed to the conception and design of the paper. AS drafted the initial manuscript, with all authors contributing to the critical revision and editing of the manuscript for intellectual content. All authors approved the final manuscript as submitted and agree to be accountable for all aspects of the work.
